# Rural Versus Urban Patients: Benchmarking the Outcomes of Patients with Acute Myocardial Infarction in Shanxi, China from 2013 to 2017

**DOI:** 10.3390/ijerph15091930

**Published:** 2018-09-05

**Authors:** Miao Cai, Echu Liu, Wei Li

**Affiliations:** 1 Department of Epidemiology and Biostatistics, College for Public Health and Social Justice, Saint Louis University, Saint Louis, MO 63103, USA; miao.cai@slu.edu; 2 Department of Health Management and Policy, College for Public Health and Social Justice, Saint Louis University, Saint Louis, MO 63103, USA; echu.liu@slu.edu; 3 Dongfang College, Zhejiang University of Finance and Economics, Hangzhou 314408, China

**Keywords:** rural-urban disparity, in-hospital mortality, out-of-pocket expense, acute myocardial infarction, electronic medical records

## Abstract

Rural-urban disparity in China attracts special international attention in view of the imbalance of economic development between rural and urban areas. However, few studies used patient level data to explore the disparity of health outcomes between rural and urban patients. This study aims to evaluate the trend of health outcomes between rural and urban patients hospitalized with acute myocardial infarction (AMI) in China. Using an electronic medical records (EMRs) database in Shanxi, China, we identified 87,219 AMI patients hospitalized between 2013 and 2017. We used multivariable binary logistic regressions and two-part models to estimate the association between region of origin (rural/urban) and two outcomes, in-hospital mortality and out-of-pocket (OOP) expenses. Rural patients were associated with lower in-hospital mortality and the adjusted Odds Ratios (ORs) were 0.173, 0.34, 0.605, 0.522, 0.556 (*p*-values < 0.001) from 2013 to 2017, respectively. For the OOP expenses, rural patients were experiencing increasing risk of having OOP expenses, with the ORs of 0.159, 0.573, 1.278, 1.281, 1.65. The coefficients for the log-linear models in the five years were 0.075 (*p* = 0.352), 0.61, 0.565, 0.439, 0.46 (*p*-values < 0.001). Policy makers in China should notice and narrow the gap of health outcomes between rural and urban patients.

## 1. Introduction

Health inequality between rural and urban areas has been a widely studied topic in the field of public health all over the world [1,2,3,4,5,6,7,8]. According to the statistics by the World Bank, the percent of people living in the rural areas has been constantly declining between 1960 and 2017, but it still remained at 45.3% in 2017 [9]. Compared to their urban counterparts, rural residents generally have limited financial incomes and access to healthcare resources, but lower levels of environmental pollution and stress. Understanding the health disparity between rural and urban areas allows health managers and policy makers to optimize healthcare resource allocation and target areas of need [10,11], and therefore improve population health outcomes.

Rural-urban disparity with regards to health outcomes in China is an established field of debate [12,13,14]. Over six million Chinese people were residing in the rural areas, accounting for about a half of the total population [15]. Eliminating the gap in health outcomes between rural and urban residents is a central target for health departments in China [13]. However, the government has been widely criticized for being limited in reducing the imbalance of healthcare resources between rural and urban residents [13,16,17]. Large academic medical centers, tertiary hospitals, and skilled medical practitioners are concentrated in urban areas, which could benefit urban residents in terms of health outcomes. A high concentration of high quality medical resources, together with advanced modern transportation, substantially improves geographic accessibility and availability of healthcare resources for urban residents in China. They can usually reach a nearby trustworthy medical provider within half an hour via public transportation, but the time cost for rural residents could double or triple in the same case.

Since the Chinese national medical reform was implemented in 2009, the healthcare coverage and accessibility have been greatly improved in both rural and urban areas of China [18,19]. However, the benefits brought by economic growth and healthcare coverage are not necessarily homogeneous across rural and urban areas. A study by Yao and his colleagues reported an overall reduction in average prescription costs, but the rural-urban disparities of parenteral administration use and expenditure per prescription were still increasing [20]. Ge and his colleagues suggested rural elderly patients had a significantly lower annual physical examination rate after adjusting for a number of covariates [21]. Another nationwide prospective study by Bragg et al. indicated significantly higher excess diabetes mortality rates among rural participants [22]. This evidence suggests that eliminating the rural-urban disparity in health resource utilization and health outcomes still has a long way to go.

To understand the trend of rural-urban disparity in China, studies with a longer sampling period are usually preferred. Several studies have presented the changing patterns of this disparity over a period of time [23,24,25,26]. However, these studies all used either survey data or aggregated registry data. To our knowledge, no study has used structured patient records to illustrate the trend of rural-urban disparity in terms of healthcare outcomes in China. To bridge the gap in existing literature, this study aims to evaluate the health outcomes between rural and urban acute myocardial infarction patients in Shanxi, China between 2013 and 2017.

## 2. Methods

### 2.1. Data Source

This study was based on the electronic medical records provided by the Health and Family Planning Commission in Shanxi, China. This database was standardized by the former Ministry of Health in 2011, and it was then required as a part of health information systems in hospitals in the entire country. The database includes over 200 patient level variables, with each patient hospitalization counting as a unique observation. These variables included patients’ demographic characteristics (age, gender, marital status, employment status), length of stay in hospital, up to 10 secondary diagnoses coded using the International Classification of Disease, Tenth Revision (ICD-10), up to seven procedures coded using the International Classification of Disease, Ninth Revision (ICD-9), gravity of disease (normal, severe or dangerous), and outcomes upon discharge (death, total medical expenses and out-of-pocket expenses). All patient unique identifiers such as names and ID card numbers were excluded prior to the authors’ access.

### 2.2. Study Population

Using patients’ primary diagnosis codes (ICD-10 code: I21), we identified 87,219 AMI patients hospitalized in Shanxi between 2013 and 2017. We also excluded patients who were less than 18 years old and those with key variables missing. This study focused on AMI patients for three reasons. First, AMI is a very commonly diagnosed disease and leads to hospitalization. It has been reported as the major cause of death among cardiovascular disease patients all over the world [27]. Second, the mortality of AMI patients has been adopted as a measure of overall medical quality by various researchers [28,29]. Third, AMI patients typically experience tremendous pain within a short amount of time and they will generally be sent to hospitals right after they exhibit symptoms. This reduces the chance of selection bias for this study.

### 2.3. Variables of Interest

The two outcomes of interest in this study were in-hospital mortality and out-of-pocket costs for AMI patients. In-hospital mortality was defined as all-cause death during the patient’s hospitalization, which was a proxy of the medical quality of that hospital. Out-of-pocket expense was defined as the direct financial expenses of the patient to the hospital, which was a proxy of the patient’s direct financial burden.

We identified patients’ region of origin (rural/urban) using a two-stage classification method. In the first stage, we used their insurance types to identify their region of origin. Patients with the insurance type of the New Cooperative Medical Scheme (NCMS) were classified as rural patients, while those with the Urban Resident-based Basic Medical Insurance Scheme (URBMI) or the Urban Employee-based Basic Medical Insurance scheme (UEBMI) were classified as urban residents. In the Chinese healthcare system, these three types of insurance are government-based and they cover over 95% of residents. URBMI and UEBMI are only available to urban residents while NCMS only covers rural residents. Therefore, these three insurance types are the golden standards to identify whether the patients were from rural or urban areas. In this first stage, we identified 83.9% of all patients’ region of origin.

In the second stage, we used regular expressions to identify the key words in patients’ current residency address. Patients whose current residency address contained county, town or village were classified as rural residents, while those whose current address contained city and district were classified as urban patients. In contrast to the first stage, this stage was a suboptimal method of classification. Some immigrant workers who came from rural areas may live and work in the urban areas, but they may not be covered by the three government-based medical insurance. For these patients, our two-stage classification method will misclassify them as urban residents. However, this bias is minimized since the first stage identified 83.9% of all patients and Shanxi province is not famous for immigrant workers.

A total of 96.7% of patients’ region of origin in this study were identified using this two-stage classification method. The patients whose region of origin were not identifiable using this classification method were excluded from this study.

### 2.4. Patient and Hospital Level Covariates

Patient level covariates included age, gender, marital status, occupation, length of stay, gravity of disease, whether percutaneous coronary intervention (PCI) was conducted, and Elixhauser score. Hospital level covariate included whether the hospital was tertiary or not. Patients’ ages were categorized into four groups (18–45, 46–65, 66–75, and over 75 years), with the 18–45 years as the reference group. Marital status was categorized into married, unmarried, widowed, divorced and other, with married as the reference. Occupation was categorized into public institution, private institution, farmer, unemployed, retired and other, with public institution as the reference. The length of stay in hospitals was categorized using the quartiles, with the lowest quartile as the reference. The gravity of disease had three categories, normal (Reference), severe and dangerous. PCIs were identified using the ICD-9 procedure codes 36.01, 36.02, 36.05, 36.06 or 36.07. Comorbidity scores have been widely applied in risk adjustment and predicting the outcomes of hospitalized patients. In this study, we used the Elixhauser comorbidity score updated by the Agency for Healthcare Research and Quality (AHRQ) in 2017 [30]. This comorbidity score was a numeric value with the greater number indicating worse comorbidities and serving as a measure of patient mix. The summary statistics stratified by years and rural-urban status are shown in Table 1.

### 2.5. Statistical Models

We used multivariable logistic regressions with binary mortality outcome in this study. In terms of patient’s OOP expense, since it was extremely right skewed and had mass density at zero, we adopted a two-part model as suggested by Deb and Norton [31]. In the first part, the probability that the patient had any OOP expenses (higher than zero) was estimated with a logistic regression using the full sample. In the second part, we used log-linear regressions to model those non-zero OOP expenses. Since the outcome OOP expenses were log transformed, the coefficients were interpreted as the ratio of the geometric mean of OOP expenses for rural patients over the geometric mean of OOP expenses for urban patients. To put it simply, the OOP expenses for rural patients were higher when the coefficient was positive, while they were lower when the coefficient was negative.

Since each independent variable may have different effects on the patients’ outcomes across the five years, we conducted the regression models separately for each year’s data. All data management, statistical modeling and data visualization were conducted in statistical computing environment R 3.4.1 [32].

## 3. Results

### 3.1. Patient Characteristics

A total of 87,219 hospital patient records with the primary diagnosis of AMI were identified in this study. These patients were admitted in 168 hospitals in Shanxi between 2013 and 2017, resulting in an average of 519.2 discharged patients in each hospital. It was to be noted that the proportion of rural patients continuously increased from 45.3% in 2013 to 58.6% in 2015. The proportion remained stable at 58% between 2015 and 2017 (Table 1).

As Table 1 shows, the mortality rates of AMI patients in rural and urban areas remained the same at 0.1% and 0.3% respectively throughout the study period. On the contrary, the OOP expenses in the two areas have been fluctuating over the five years. The percent of urban patients who had no OOP expenses had been increasing while a reversed trend could be observed among rural patients. The median of non-zero OOP expenses among rural patients was less than those among urban patients in 2013. However, starting from 2014, rural patients had much more OOP expenses than their urban counterparts as shown by the medians, although the gap had been shrunk over the four years. The sharpest contrast between the rural and urban patients in Table 1 was their occupation. The proportion of rural patients who were farmers consistently increased from 67.4% in 2013 to 83.6% in 2017. However, the same measure for urban patients decreased from 16.5% in 2013 to 6.7% in 2017 and never exceeded 20%. Table 1 also showed that rural patients consistently had less severe disease than urban patients, based on the percent of rural patients over 75 years old, the percent of rural patients graded as dangerous, and the average Elixhauser scores of rural patients.

### 3.2. In-Hospital Mortality

**Figure 1:**
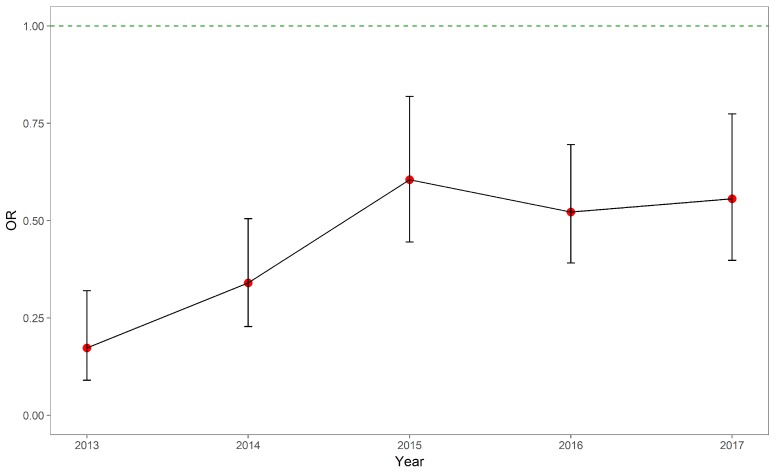
Odds ratios of rural patients with regard to in-hospital mortality, 2013–2017.

As shown in Table 2, the odds ratios of rural AMI patients were consistently less than 1 across the study period, after adjusting for demographics, length of stay, gravity of disease, Elixhauser score, PCI and level of hospital. The trend can also be observed from Figure 1. Although the ORs were constantly significant and less than 1, the gap between rural and urban AMI patients had been shrunk across the five years. Patients’ marital status and occupation were not significantly associated with the patients’ probability of in-hospital death since most of these categories were not significant at the level of 0.05.

### 3.3. OOP Expenses

**Figure 2:**
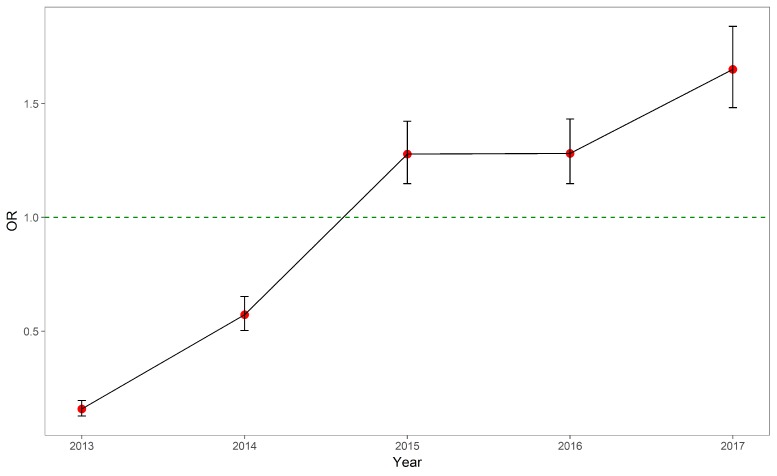
Odds ratios of rural patients with regard to whether out-of-pocket expenses were zero, 2013–2017.

**Figure 3:**
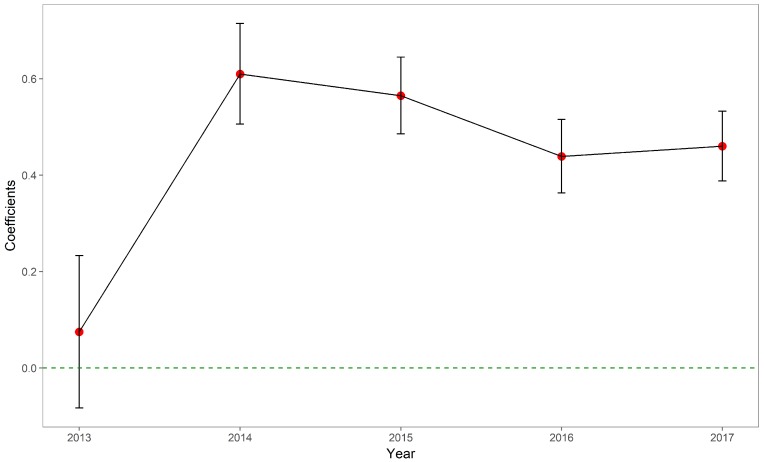
Coefficients of rural patients in terms of non-zero OOP expenses, 2013–2017.

In contrast to the in-hospital mortality, the coefficients for OOP expenses experienced more fluctuations. Table 3 and Figure 2 displayed the trend of ORs to have non-zero OOP expense among rural AMI patients compared with urban AMI patients. The probabilities to have non-zero OOP expenses among rural AMI patients had been continuously increasing compared with their urban counterparts. Moreover, the non-zero OOP expenses among rural patients had been higher than their urban counterparts as shown in Table 4 and Figure 3. The differences in non-zero expenses had been reduced over the study period, as indicated by the absolute distance from the point estimates to the reference line.

## 4. Discussion

The administrative hospitalized AMI patient data in this study indicated that the disparity of health outcomes between rural and urban patients have been reduced from 2013 to 2017. Despite the closing gap, the in-hospital mortality rates of rural patients were consistently lower than those of urban patients after adjusting for a set of patient and hospital level variables. Rural AMI patients had an increasing risk of having OOP expenses compared with their urban counterparts. The adjusted non-zero OOP expenses of rural AMI patients had been consistently higher than urban patients. In view of the fact that cardiovascular disease is the leading cause of death globally and in China [27,33], these trends and changing patterns deserve special attention from health policy makers and administrators in China.

Our results highlighted that urban patients had higher in-hospital mortality rates after controlling for the covariates. These results agree with several previous studies [12,34,35]. For example, a study based on China Health and Nutrition Survey between 1997 and 2006 suggested that rural residents had better overall health status and healthcare utilization [12]. Another nationwide sample of hospitalized Chinese AMI patients in 2015 found that patients covered by the NCMS had significantly lower adjusted in-hospital mortality rates than patients with the UEBMI [34]. These results, together with this study, all signify a higher mortality among urban residents, compared to rural residents.

There are multiple reasons for the result that rural patients had lower adjusted in-hospital mortality rates than urban patients. First, high pollution level and unhealthy lifestyle in urban areas may explain this disparity. With rapid economic development in recent years, urban residents are subject to higher air and water pollution compared with rural residents [14]. Second, Western-style diets have been widely adopted and accepted as a routine eating pattern in the urban areas, but it is less far-reaching in the rural areas because of lower economic growth and less population density [36]. Lee and her colleagues reported that Westernized fast food was linked with poorer psychological health status among the Chinese population [37]. With Western-style diets being more and more integrated into daily lives of urban residents, we are expecting a worrying enlarging gap of mortality between rural and urban patients in the future. Third, inadequate patient-mix adjustment may be another reason why rural patients had lower adjusted in-hospital mortality rates than urban patients in this study. Based on previous analysis that rural residents have less environmental population and Western-style diets, we believe that rural patients are less sick than urban patients. Although we used the Elixhauser comorbidity score developed by the AHRQ in 2017, the score was actually developed based on the American population [30]. No study has yet validated the application of Elixhauser comorbidity score on the Asian population or developed a comorbidity score that specifically targets the Asian population.

The probability that rural AMI patients had OOP expenses revealed an unceasingly increasing pattern compared to urban patients. This trend agrees with several studies from other developing countries. For example, Garg and Karan reported that residents in rural areas had a higher proportion of OOP expenditure than their urban counterparts in India [38]. Another study reported substantial OOP expenditure for women delivery in rural Tanzania, although there was no comparison between rural and urban women [39]. It seems unusual that over 60% of AMI patients in this study had no OOP expenditure during their hospitalization. This mass zero density may be associated with local medical insurance policies, with no costs for veterans and some civil servants. The most concerning result in this study is that the OOP expenses of rural residents have been consistently higher than those of urban residents. This may stem from different reimbursement rates of rural and urban insurance schemes and local policies [40,41]. The reimbursement level of the NCMS for rural residents was 10% lower and its coverage of medical service was narrower than those of either URBMI and UEBMI for urban residents or employees [41], since the NCMS funds are pooled at the county level while the UEBMI and URBMI fund are pooled at the city level. The reimbursement and benefit packages were decided by local policy makers, which could contribute to the enlarging gap of OOP expenses between rural and urban patients. The fragmentation and inequity in social health insurances in China have been widely criticized recently [41,42,43]. Researchers have been calling for an integration into a universal healthcare insurance scheme in China. However, the integration process is difficult and slow, mostly because of diverse funding levels and a lack of national guidelines [41]. The ideal pattern should be a narrowing trend between rural and urban residents. Additionally, rural residents should have less OOP expenses in view of their lower incomes. The Chinese government aims to achieve universal health coverage by 2020, but the progress seems to be behind.

The other interesting result in this study is the level of hospitals. The ORs of tertiary hospitals maintained a growing trend and it exceeded 1 in 2017, which indicated that tertiary hospitals had higher adjusted mortality rates than secondary hospitals. Similar results have been revealed in other studies [44,45]. Tertiary hospitals in China are large academic centers with the most skillful and experienced doctors and nurses, and they are expected to provide the best medical services in the country. However, our study suggested that tertiary hospitals underperform with regard to in-hospital mortality. The underlying reason behind this unexpected result in not clear, but patient overload may have contributed to it. Doctors and nurses in China, especially in tertiary hospitals, have constantly been experiencing patient overload. On average, a doctor in China will have to serve 735 patients annually, which is substantially higher than the number in Western countries (280 to 640 patients) [46]. Job overload and burnout could have threatened the quality of care in Chinese tertiary hospitals.

We noticed that the number of AMI patients experienced an expansion between 2013 and 2015, but the trend seemed to disappear after 2015. This is because the EMR system in Shanxi was initiated and piloted in a few large medical centers in 2013, and the number of hospitals applying this EMR system continued to increase until 2015, when the EMR system was applied to hospitals in the entire province. Although the number of AMI patients in 2013 and 2014 was relatively small compared with that in 2015, 2016 and 2017, the study team could still have sufficient statistical power to make inference about the parameters of rural-urban disparity with thousands of patient records.

This study has several limitations. First, since this study is based on administrative medical records, it does not contain information of patients’ follow-ups. Patient outcomes can only be observed during their hospitalization, and we do not have their follow-ups after discharge. Second, the patients were divided into rural or urban regions according to their insurance status and current residency address. However, the second stage classification may lead to misclassification bias. The second stage was a suboptimal method of classification since some immigrant workers who came from rural areas may live and work in the urban areas, but they may not be covered by the insurance mentioned in this paper. For these patients, our two-stage classification method will misclassify them as urban residents. A more robust method may be using the patients’ registry information, which were not collected in our database. Third, as an observational study, this study is subject to omitted variable bias. Door-to-balloon time is a crucial variable that can influence the outcomes of patients [47], but it is not recorded in hospital EMRs in Shanxi, China.

## 5. Conclusions

The disparities with regard to in-hospital mortality and OOP expenses between rural and urban patients are shrinking in China. The adjusted in-hospital mortality rates of rural patients were significantly lower than those of urban patients, but the adjusted OOP expenses of rural patients were significantly higher than those of urban patients. Policy makers are recommended to narrow the gap of health outcomes between rural and urban patients.

## Figures and Tables

**Table 1:** Descriptive statistics of sample acute myocardial infarction patient characteristics, 2013–2017.

	2013		2014		2015		2016		2017	
Urban/Rural	Urban	Rural	Urban	Rural	Urban	Rural	Urban	Rural	Urban	Rural
***n* (%)**	2243 (54.7)	1854 (45.3)	7073 (50.4)	6966 (49.6)	9092 (41.4)	12,882 (58.6)	10,349 (41.7)	14,458 (58.3)	9233 (41.4)	13,069 (58.6)
**Death**	0.03 (0.18)	0.01 (0.10)	0.03 (0.17)	0.01 (0.12)	0.03 (0.17)	0.01 (0.12)	0.03 (0.18)	0.01 (0.12)	0.03 (0.17)	0.01 (0.12)
**Out-of-pocket expenses**										
% of 0 values	68.9	86.7	72.4	76.4	71.8	76.1	76.2	80.1	71.8	75.3
median	12,331	10,953	12,591	22,714	12,701	17,602	11,939	13,694	12,582	15,664
1st quartile	4652	6168	4796	9260	4677	8216	4911	7749	5542	8015
3rd quartile	24,837	33,465	22,864	45,539	25,065	42,488	22,304	35,545	24,338	37,474
**Female**	0.22 (0.42)	0.29 (0.45)	0.22 (0.41)	0.29 (0.45)	0.22 (0.41)	0.31 (0.46)	0.21 (0.41)	0.30 (0.46)	0.21 (0.41)	0.30 (0.46)
**Age (%)**										
18–45	226 (10.1)	246 (13.3)	651 (9.2)	892 (12.8)	831 (9.1)	1392 (10.8)	910 (8.8)	1476 (10.2)	711 (7.7)	1252 (9.6)
46–65	993 (44.3)	981 (52.9)	3205 (45.3)	3836 (55.1)	4145 (45.6)	6552 (50.9)	4632 (44.8)	7390 (51.1)	4143 (44.9)	6755 (51.7)
66–75	559 (24.9)	386 (20.8)	1592 (22.5)	1457 (20.9)	2021 (22.2)	3033 (23.5)	2248 (21.7)	3402 (23.5)	2069 (22.4)	3190 (24.4)
75+	465 (20.7)	241 (13.0)	1625 (23.0)	781 (11.2)	2095 (23.0)	1905 (14.8)	2559 (24.7)	2190 (15.1)	2310 (25.0)	1872 (14.3)
**Marriage (%)**										
Married	2101 (93.7)	1680 (90.6)	6578 (93.0)	6439 (92.4)	8355 (91.9)	11,853 (92.0)	9525 (92.0)	13,343 (92.3)	8330 (90.2)	11,932 (91.3)
Unmarried	26 (1.2)	79 (4.3)	94 (1.3)	213 (3.1)	94 (1.0)	216 (1.7)	97 (0.9)	253 (1.7)	223 (2.4)	360 (2.8)
Widowed	60 (2.7)	51 (2.8)	194 (2.7)	156 (2.2)	372 (4.1)	471 (3.7)	401 (3.9)	496 (3.4)	373 (4.0)	431 (3.3)
Divorced	48 (2.1)	23 (1.2)	167 (2.4)	112 (1.6)	159 (1.7)	145 (1.1)	173 (1.7)	155 (1.1)	176 (1.9)	140 (1.1)
Other	8 (0.4)	21 (1.1)	40 (0.6)	46 (0.7)	112 (1.2)	197 (1.5)	153 (1.5)	211 (1.5)	131 (1.4)	206 (1.6)
**Occupation (%)**										
Public institution	194 (8.6)	33 (1.8)	902 (12.8)	127 (1.8)	1273 (14.0)	145 (1.1)	1421 (13.7)	157 (1.1)	1258 (13.6)	105 (0.8)
Private institution	617 (27.5)	97 (5.2)	1989 (28.1)	346 (5.0)	2041 (22.4)	386 (3.0)	2321 (22.4)	462 (3.2)	1998 (21.6)	448 (3.4)
Farmer	370 (16.5)	1249 (67.4)	385 (5.4)	5553 (79.7)	634 (7.0)	10,526 (81.7)	680 (6.6)	12,158 (84.1)	621 (6.7)	10,928 (83.6)
Jobless	110 (4.9)	47 (2.5)	383 (5.4)	223 (3.2)	560 (6.2)	236 (1.8)	640 (6.2)	294 (2.0)	563 (6.1)	268 (2.1)
Retired	601 (26.8)	90 (4.9)	2581 (36.5)	292 (4.2)	3437 (37.8)	350 (2.7)	4203 (40.6)	412 (2.8)	3872 (41.9)	286 (2.2)
Other	351 (15.6)	338 (18.2)	833 (11.8)	425 (6.1)	1147 (12.6)	1239 (9.6)	1084 (10.5)	975 (6.7)	921 (10.0)	1034 (7.9)
**Length of stay (%)**										
1st quartile	432 (19.3)	484 (26.1)	1380 (19.5)	1768 (25.4)	2082 (22.9)	3882 (30.1)	2519 (24.3)	4470 (30.9)	2370 (25.7)	4458 (34.1)
2nd quartile	536 (23.9)	521 (28.1)	1807 (25.5)	2018 (29.0)	2467 (27.1)	3665 (28.5)	3076 (29.7)	4534 (31.4)	3007 (32.6)	4340 (33.2)
3rd quartile	525 (23.4)	406 (21.9)	1650 (23.3)	1544 (22.2)	2074 (22.8)	2885 (22.4)	2180 (21.1)	3037 (21.0)	1899 (20.6)	2454 (18.8)
4th quartile	750 (33.4)	443 (23.9)	2236 (31.6)	1636 (23.5)	2469 (27.2)	2450 (19.0)	2574 (24.9)	2417 (16.7)	1957 (21.2)	1817 (13.9)
**Gravity of disease (%)**										
Dangerous	488 (21.8)	337 (18.2)	1719 (24.3)	1181 (17.0)	2106 (23.2)	2253 (17.5)	2193 (21.2)	2843 (19.7)	1915 (20.7)	2560 (19.6)
Severe	628 (28.0)	499 (26.9)	1873 (26.5)	2141 (30.7)	2074 (22.8)	2771 (21.5)	2610 (25.2)	3642 (25.2)	2321 (25.1)	3267 (25.0)
Normal	1127 (50.2)	1018 (54.9)	3481 (49.2)	3644 (52.3)	4912 (54.0)	7858 (61.0)	5546 (53.6)	7973 (55.1)	4997 (54.1)	7242 (55.4)
**Percutaneous coronary intervention**	0.14 (0.34)	0.15 (0.36)	0.17 (0.38)	0.15 (0.36)	0.19 (0.39)	0.13 (0.33)	0.23 (0.42)	0.17 (0.38)	0.31 (0.46)	0.24 (0.43)
**Tertiary hospitals (%)**	2215 (98.8)	1840 (99.2)	7013 (99.2)	6841 (98.2)	7773 (85.5)	7855 (61.0)	8634 (83.4)	8889 (61.5)	7859 (85.1)	8195 (62.7)
**Elixhauser score**	4.91 (5.90)	4.99 (5.68)	6.10 (6.11)	6.08 (5.91)	6.46 (6.21)	5.90 (6.26)	7.17 (6.18)	6.46 (6.17)	7.42 (6.30)	6.63 (6.28)

The percent of 0 values for OOP expenses were based on the full sample, while the median, the 1st and the 3rd quartiles were based on non-zero OOP expenses.

**Table 2:** Logistic regression results for in-hospital death, 2013–2017.

	2013		2014		2015		2016		2017	
	OR	*p*-Value	OR	*p*-Value	OR	*p*-Value	OR	*p*-Value	OR	*p*-Value
**(Intercept)**	0.211	0.097	0.04	<0.001	0.035	<0.001	0.017	<0.001	0.009	<0.001
**Rural (Ref. = Urban)**	0.173	<0.001	0.34	<0.001	0.605	0.001	0.522	<0.001	0.556	0.001
**Female (Ref. = Male)**	1.82	0.023	1.153	0.305	1.164	0.179	1.404	0.001	1.392	0.003
**Age (Ref. = 18–45)**										
46–65	0.661	0.373	2.291	0.038	1.398	0.218	1.662	0.067	1.313	0.348
66–75	0.961	0.935	4.059	0.001	2.915	<0.001	2.863	<0.001	2.069	0.014
≥76	2.344	0.072	6.347	<0.001	3.307	<0.001	4.358	<0.001	3.08	<0.001
**Marriage (Ref. = Married)**										
Unmarried	2.875	0.067	1.363	0.483	0.768	0.61	0.478	0.215	1.059	0.886
Widowed	0.376	0.136	1.119	0.686	1.22	0.297	1.015	0.93	1.293	0.171
Divorced	3.281	0.014	1.941	0.012	2.09	0.006	0.691	0.308	0.854	0.662
Other	5.028	0.141	1.642	0.438	0.882	0.767	1.501	0.18	2.387	0.003
**Occupation (Ref. = Public institution)**										
Private institution	0.824	0.729	1.608	0.193	0.877	0.627	1.107	0.73	1.908	0.104
Farmer	1.411	0.526	2.382	0.022	0.67	0.128	1.174	0.575	2.145	0.055
Jobless	0.478	0.305	0.731	0.509	0.738	0.357	1.209	0.559	2.059	0.09
Retired	1.082	0.882	2.273	0.017	1.399	0.15	2.052	0.006	3.615	0.001
Other	0.556	0.32	0.891	0.776	0.604	0.065	1.082	0.788	1.459	0.361
**Length of stay (Ref. 1st quartile)**										
2nd quartile	0.112	<0.001	0.136	<0.001	0.13	<0.001	0.103	<0.001	0.116	<0.001
3rd quartile	0.023	<0.001	0.062	<0.001	0.085	<0.001	0.048	<0.001	0.076	<0.001
4th quartile	0.107	<0.001	0.118	<0.001	0.149	<0.001	0.119	<0.001	0.225	<0.001
**Gravity of disease (Ref. Normal)**										
Dangerous	1.655	0.041	1.707	<0.001	1.553	<0.001	2.305	<0.001	2.323	<0.001
Severe	0.48	0.032	0.825	0.23	0.866	0.308	0.968	0.794	0.97	0.831
**PCI**	0.566	0.292	0.137	<0.001	0.296	<0.001	0.339	<0.001	0.423	<0.001
**Level of hospitals (Ref. = Secondary)**										
Tertiary	0.377	0.181	0.352	0.001	0.953	0.686	1.185	0.11	1.474	0.002
**Elixhauser score**	1.053	0.002	1.023	0.011	1.023	0.002	1.018	0.008	1.016	0.025

**Table 3:** Logistic regression results for whether OOP expenses were zero, 2013–2017.

	2013		2014		2015		2016		2017	
	Coefficient	*p*-Value	Coefficient	*p*-Value	Coefficient	*p*-Value	Coefficient	*p*-Value	Coefficient	*p*-Value
**(Intercept)**	0.33	0.012	0.515	0.001	0.163	<0.001	0.147	<0.001	0.154	<0.001
**Rural (Ref. = Urban)**	0.159	<0.001	0.573	<0.001	1.278	<0.001	1.281	<0.001	1.65	<0.001
**Female (Ref. = Male)**	0.895	0.282	0.968	0.524	1.013	0.753	1.082	0.056	1.147	0.001
**Age (Ref. = 18–45)**										
46–65	1.006	0.964	0.965	0.588	1.003	0.956	1.015	0.803	1.184	0.006
66–75	1.208	0.22	1.083	0.298	1.088	0.191	1.001	0.991	1.278	<0.001
75+	1.508	0.014	0.999	0.994	1.009	0.898	1.158	0.042	1.405	<0.001
**Marriage (Ref. = Married)**										
Unmarried	0.151	<0.001	0.343	<0.001	0.627	0.004	1.114	0.437	0.882	0.219
Widowed	0.839	0.509	0.975	0.849	0.646	<0.001	0.753	0.005	0.676	<0.001
Divorced	0.24	0.003	0.254	<0.001	0.691	0.015	0.973	0.852	1.91	<0.001
Other	0.206	0.124	0.163	<0.001	0.147	<0.001	0.223	<0.001	0.34	<0.001
**Occupation (Ref. = Public institution)**										
Private institution	2.46	<0.001	0.878	0.11	0.871	0.059	1.339	<0.001	1.317	<0.001
Farmer	6.43	<0.001	1.134	0.175	0.575	<0.001	0.776	0.003	0.585	<0.001
Jobless	2.625	0.001	1.101	0.411	0.902	0.303	1.486	<0.001	1.257	0.026
Retired	1.011	0.96	0.549	<0.001	0.603	<0.001	1.188	0.025	1.037	0.634
Other	1.784	0.008	0.203	<0.001	0.348	<0.001	0.318	<0.001	0.551	<0.001
**Length of stay (Ref. 1st quartile)**										
2nd quartile	1.261	0.054	1.279	<0.001	1.085	0.077	0.914	0.044	0.766	<0.001
3rd quartile	1.279	0.047	1.383	<0.001	1.142	0.006	0.854	0.002	0.704	<0.001
4th quartile	1.093	0.452	1.192	0.003	1.328	<0.001	0.977	0.637	0.918	0.079
**Gravity of disease (Ref. Normal)**										
Dangerous	0.984	0.883	1.207	<0.001	1.368	<0.001	1.598	<0.001	1.504	<0.001
Severe	1	0.997	1.058	0.236	1.075	0.078	0.623	<0.001	0.62	<0.001
**PCI**	3.083	<0.001	1.923	<0.001	2.875	<0.001	4.389	<0.001	2.886	<0.001
**Level of hospital (Ref. = Secondary hospital)**										
Tertiary	0.508	0.077	0.638	0.008	2.375	<0.001	1.74	<0.001	2.004	<0.001
**Elixhauser score**	0.966	<0.001	1.027	<0.001	1.005	0.048	0.97	<0.001	0.985	<0.001

**Table 4:** Log-linear model results for non-zero OOP expenses, 2013–2017.

	2013		2014		2015		2016		2017	
	OR	*p*-Value	OR	*p*-Value	OR	*p*-Value	OR	*p*-Value	OR	*p*-Value
**(Intercept)**	8.278	<0.001	7.913	<0.001	7.91	<0.001	7.998	<0.001	7.883	<0.001
**Rural (Ref. = Urban)**	0.075	0.352	0.61	<0.001	0.565	<0.001	0.439	<0.001	0.46	<0.001
**Female (Ref. = Male)**	−0.034	0.69	−0.101	0.012	−0.072	0.017	−0.01	0.741	−0.006	0.845
**Age (Ref. = 18–45)**										
46–65	−0.204	0.054	−0.021	0.69	−0.111	0.006	−0.07	0.094	−0.143	0.001
66–75	−0.25	0.042	−0.237	<0.001	−0.222	<0.001	−0.182	<0.001	−0.189	<0.001
75+	−0.533	<0.001	−0.493	<0.001	−0.474	<0.001	−0.379	<0.001	−0.385	<0.001
**Marriage (Ref. = Married)**										
Unmarried	0.9	0.065	0.169	0.336	0.009	0.945	−0.158	0.107	−0.424	<0.001
Widowed	0.281	0.185	−0.168	0.109	0.01	0.9	−0.183	0.019	−0.051	0.499
Divorced	0.045	0.917	0.026	0.894	0.082	0.486	0.074	0.476	−0.108	0.177
Other	−0.071	0.941	0.007	0.987	−0.015	0.956	−0.056	0.799	−0.396	0.022
**Occupation (Ref. = Public institution)**										
Private institution	−0.586	0.001	−0.603	<0.001	−0.349	<0.001	−0.196	<0.001	−0.055	0.314
Farmer	0.582	0.001	−0.106	0.145	0.065	0.243	0.046	0.434	0.205	<0.001
Jobless	−0.195	0.407	−0.142	0.107	0.039	0.572	−0.046	0.526	0.16	0.026
Retired	−0.199	0.292	-0.362	<0.001	−0.045	0.374	0.01	0.859	0.069	0.204
Other	−0.015	0.935	−0.214	0.048	0.021	0.769	0.042	0.607	0.313	<0.001
**Length of stay (Ref. 1st quartile)**										
2nd quartile	0.4	<0.001	0.501	<0.001	0.509	<0.001	0.396	<0.001	0.41	<0.001
3rd quartile	0.584	<0.001	0.689	<0.001	0.749	<0.001	0.625	<0.001	0.577	<0.001
4th quartile	1.047	<0.001	1.139	<0.001	1.224	<0.001	1.115	<0.001	1.035	<0.001
**Gravity of disease**										
Dangerous	−0.201	0.019	0.079	0.055	0.005	0.855	0.213	<0.001	0.091	0.001
Severe	−0.171	0.028	−0.027	0.465	−0.1	0.001	−0.131	<0.001	−0.094	0.003
**PCI**	1.233	<0.001	0.887	<0.001	0.752	<0.001	0.844	<0.001	0.869	<0.001
**Level of hospital (Ref. = Secondary hospital)**										
Tertiary	0.418	0.173	0.875	<0.001	0.722	<0.001	0.535	<0.001	0.603	<0.001
**Elixhauser score**	−0.01	0.076	0.005	0.045	0.008	<0.001	−0.002	0.274	0.011	<0.001

## Abbreviations

The following abbreviations are used in this manuscript:

AMI	Acute Myocardial Infarction
EMR	Electronic Medical Records
ICD-9	The International Classification of Disease, Ninth Revision
ICD-10	The International Classification of Disease, Tenth Revision
NCMS	New Cooperative Medical Scheme
OR	Odds Ratio
OOP	Out-of-pocket
PCI	Percutaneous Coronary Intervention
URBMI	Urban Resident-based Basic Medical Insurance Scheme
UEBMI	Urban Employee-based Basic Medical Insurance Scheme
